# The Role of Gut Microbiota and Circadian Rhythm Oscillation of Hepatic Ischemia–Reperfusion Injury in Diabetic Mice

**DOI:** 10.3390/biomedicines12010054

**Published:** 2023-12-25

**Authors:** Juan Li, Yanbo Liu, Yijing Li, Tianning Sun, Hongbing Xiang, Zhigang He

**Affiliations:** Hubei Key Laboratory of Geriatric Anesthesia and Perioperative Brain Health, Wuhan Clinical Research Center for Geriatric Anesthesia, Department of Anesthesiology, Tongji Hospital, Tongji Medical College, Huazhong University of Science and Technology, Wuhan 430030, China; ethereal13@163.com (J.L.); bruce_lyb123@hotmail.com (Y.L.); lyj1873600@163.com (Y.L.); tianning.sun97@gmail.com (T.S.)

**Keywords:** diabetes, hepatic ischemia–reperfusion injury, circadian rhythm oscillation, intestinal microbiota, gut microbiota transplantation

## Abstract

Circadian rhythm oscillation and the gut microbiota play important roles in several physiological functions and pathology regulations. In this study, we aimed to elucidate the characteristics of diabetic hepatic ischemia–reperfusion injury (HIRI) and the role of the intestinal microbiota in diabetic mice with HIRI. Hepatic ischemia–reperfusion injury surgery was performed at ZT0 or ZT12. The liver pathological score and the serum levels of alanine aminotransferase (ALT) and aspartate aminotransferase (AST) were analyzed to evaluate liver injury. We conducted an FMT experiment to examine the role of intestinal microbiota in diabetic mice with HIRI. The 16S rRNA gene sequencing of fecal samples was performed for microbial analysis. Our results showed that hyperglycemia aggravated HIRI in diabetic mice, but there was no diurnal variation seen in diabetic HIRI. We also demonstrated that there were significant alterations in the gut microbiota composition between the diabetic and control mice and that gut microbiota transplantation from diabetic mice had obvious harmful effects on HIRI. These findings provide some useful information for the future research of diabetic mice with HIRI.

## 1. Introduction

The circadian rhythm plays a crucial role in mammalian behavior and metabolic processes, which is a defining feature of mammalian metabolism [1]. Circadian rhythm oscillation has also been linked to various physiological functions and pathology regulation [2,3]. The gut microbiota has been shown to influence human metabolism and can affect the occurrence and development of metabolic diseases [4,5,6,7,8]. Studies have demonstrated that intestinal flora follow the endogenous circadian rhythm, and that a circadian rhythm disturbance can significantly affect the composition of the gut microbiota. In contrast, the change in microbial metabolites can also regulate the transcriptional expression of the host circadian gene [9,10]. This suggests a complex relationship between the gut microbiota and the circadian rhythm of the host metabolism.

Hepatic ischemia–reperfusion injury (HIRI) occurs during liver resection, liver transplantation, and other liver surgeries. It is inevitable and is becoming one of the leading causes of post-surgery hepatic dysfunction, often resulting in morbidity and mortality [11]. The liver is usually more susceptible to ischemia–reperfusion injury due to the damaging effects of diabetes-related microcirculatory disease and inflammatory response, and hyperglycemia has been reported to exacerbate ischemia–reperfusion injury in the liver [12,13]. With the rapid increase in the incidence of diabetes and its implications, there is a growing need to pay attention to HIRI in patients with diabetes. A previous study reported the mechanism of the diurnal variation of HIRI in mice [14], but it needs further research. The features and mechanisms of the diurnal variation of HIRI in the diabetic host remain unclear and also require further investigation. Previous studies have shown that the intestinal microbiota is associated with obesity, diabetes, inflammatory bowel disease, and other diseases of the human host. As the important component of the ‘gut-liver axis’, it directly or indirectly affects the structure and function of the liver and is involved in the pathogenesis of non-alcoholic fatty liver disease, hepatic ischemia–reperfusion injury, and liver failure in cirrhosis [14,15,16].Studies have reported that in the early stage of reperfusion, the analysis of stool samples exhibits some degree of gut dysbiosis and increases translocation. In turn, an intestinal flora imbalance can exacerbate liver injury and promote chronic inflammatory disease of the liver [17,18]. Nevertheless, the gut microbiota also plays an important role in the pathogenesis of diabetes, and its study is a core step in understanding the relationship.

However, whether HIRI shows circadian rhythm in diabetic patients, or is related to the gut microbiota and its mechanism in diabetic HIRI, remains to be examined.

In this study, we showed that hyperglycemia aggravated HIRI in diabetic mice, but there was no diurnal variation seen in the diabetic HIRI. We conducted an FMT experiment to examine the role of the intestinal microbiota in diabetic mice with HIRI. The gut microbiota transplantation from diabetic mice had obvious harmful effects on HIRI. There were significant alterations in the gut microbiota composition between the diabetic and control mice based on 16S rRNA gene sequencing of fecal samples. Our results enhance our understanding of the characteristics of diabetic HIRI and the role of intestinal microbiota in diabetic mice with HIRI. 

## 2. Materials and Methods

### 2.1. Animal Care

Male C57BL/6J mice, aged 6–8 weeks, were purchased from the Beijing Vital River Laboratory Animal Technology Co., Ltd. (Beijing, China). They were kept in standard housing conditions (22 ± 1 °C room temperature with a relative humidity of 55 ± 5%) and a 12/12 h light/dark cycle (08:00, light on; 20:00, light off). The protocol of all experiments was approved by the Animal Care and Use Committee of Tongji Hospital, and Tongji Medical College approved the current study (no. TJH-202206014).

### 2.2. Mouse Diabetes Model

The mice were randomly divided into a diabetic group and a normal group. Firstly, the blood glucose concentration of the two groups of mice was measured as the baseline glucose serum concentration. Then, the diabetic mice were fed a high-fat (proteins 20%, carbohydrates as sucrose, maltodextrin 20%, lipids 60% of total kcal) Research Diet. After four weeks of adaption, streptozotocin (80 mg/kg in citrate buffer; Sigma, St. Louis, MO, USA) was given through intraperitoneal injection for three consecutive days to induce type 2 diabetes in mice. The vehicle control group (normal group) was subjected to the same intraperitoneal injection procedure, but with sodium citrate buffer. Tail vein blood was collected one week post injection. Mice with blood glucose levels >16.7 mmol/L for 3 consecutive days were considered as diabetes onset and selected for further study. Blood samples were collected from the tail vein and placed on the target area of the testing strip using an Accu-Chek blood glucose monitor for blood glucose concentration (Accu-Chek Performa Strips and Glucometer, Roche Diagnostics, Indianapolis, IN, USA).

### 2.3. Model of Warm Liver IRI

Hepatic ischemia–reperfusion injury surgery was performed at ZT0 (08:00, light on) or ZT12 (20:00, light off). Both the diabetic and normal mice were randomly divided into four groups (normal mice: ZT0-HIRI, *n* = 6; ZT12-HIRI, *n* = 6; ZT0-Sham, *n* = 6; ZT12-Sham, *n* = 6, diabetic mice: ZT0-HIRI, *n* = 6; ZT12-HIRI, *n* = 6; ZT0-Sham, *n* = 6; ZT12-Sham, *n* = 6). Applying a 70% liver ischemia model, the mice were anesthetized with 1% pentobarbital sodium (50 mg/kg, intraperitoneal) and then placed on an operating table. The abdominal skin was disinfected and a median incision made to expose the liver area, and then the vascular structures to the left and median lobe were identified. The blood supply to the left and median hepatic lobes was blocked for 90 min using an artery clamp. Then, the clamp was removed for 6 h of reperfusion. The hepatic ischemia–reperfusion surgery was performed according to the method previously described [14]. Then, the mice were euthanized and the serum and tissues were collected for further analysis. There was no vascular occlusion in the sham-controlled mice. During the operation, the mice were kept warm, and the surgical incision was treated with analgesia after the operation. 

### 2.4. Pseudo Germ-Free Mice Experiment and Fecal Microbiota Transplantation

C57BL/6J mice were administered with ABX (neomycin sulfate (200 mg/kg), metronidazole (200 mg/kg), ampicillin (200 mg/kg), and vancomycin (100 mg/kg) via oral gavage once a day for 4 days to eliminate the gut microbiota and induce the pseudo germ-free mice. Then, hepatic ischemia–reperfusion injury surgery was performed on the pseudo germ-free diabetic mice and normal glucose mice. Some pseudo germ-free diabetic mice and normal mice underwent the sham operation.

Fecal microbiota transplantation (FMT) was performed as described previously [14]. Briefly, donor mice feces from the diabetic and normal groups were collected and resuspended in phosphate-buffered saline (PBS) to a concentration of 0.125 g/mL. This suspension of two groups was administered to pseudo germ-free diabetic mice via oral gavage (0.15 mL) daily for 3 days. After 3 days, those mice underwent hepatic I/R surgery and were euthanized for further analysis.

### 2.5. Bacterial Composition Analysis

The fecal samples were collected at ZT0 and ZT12, and then stored at −80 °C. DNA was extracted using a DNA Extraction Kit (Tiangen Biotechnology Co., Ltd., Beijing, China). Then, 16 S rRNA sequencing of the microbiota was performed at OEBiotech Co. Ltd., Shanghai, China. The V3–V4 variable regions of the 16S rRNA genes were amplified with universal primers 343 F and 798 R, V3V4 forward primer: 343F TACGGRAGGCAGCAG; reverse primer: 798R AGGGTATCTAATCCT. The purified PCR products were used for sequencing. The software and platform accessed on 17 November 2022 (https://cloud.oebiotech.cn/task/) provided by the company were used for further bioinformatic analysis of the raw data.

### 2.6. Blood Sample and H&E Staining

The mice were deeply anesthetized with sodium pentobarbital (i.p. 50 mg/kg), and then immediately decapitated for blood collection. The levels of alanine aminotransferase (ALT) and aspartate aminotransferase (AST) in the upper serum were analyzed at the clinical laboratory of Tongji Hospital. The left lobe of the liver was fixed in a 4% neutral formaldehyde solution. The liver specimens were treated with standard HE procedures, including steps such as dehydration, embedding, sectioning, and tissue staining. The staining was scanned on each slide and the degree of liver damage graded using the Suzuki score. According to the Suzuki score criteria, the histological injury score of each sample was expressed as the sum of the individual scores for three different parameters: congestion (none = 0, minimal = 1, mild = 2, moderate = 3, severe = 4), vacuolization (none = 0, minimal = 1, mild = 2, moderate = 3, severe = 4), and necrosis (none = 0, single cell necrosis = 1, <30% = 2, 30−60% = 3, >60% = 4). The scores for each parameter ranged from 0 to 4, with a maximum possible score of 12 [19].

### 2.7. Experimental Design

**Figure 1:**
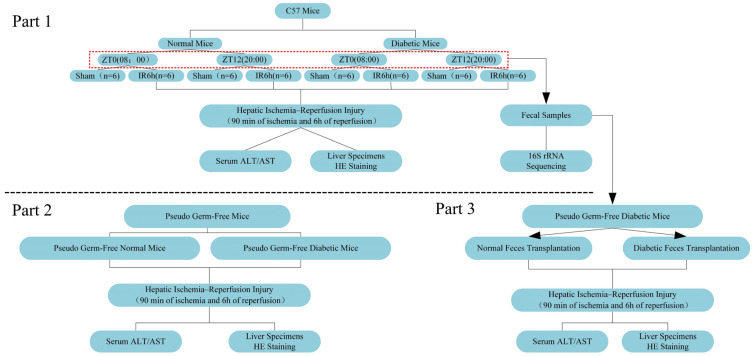
Schematic illustration of the experimental design.

Experiment 1: The mice were randomly divided into the diabetic and normal group, and then we established the model of diabetic mice. Both the diabetic and normal mice were randomly divided into four groups (normal mice: ZT0-HIRI, *n* = 6; ZT12-HIRI, *n* = 6; ZT0-Sham, *n* = 6; ZT12-Sham, *n* = 6, diabetic mice: ZT0-HIRI, *n* = 6; ZT12-HIRI, *n* = 6; ZT0-Sham, *n* = 6; ZT12-Sham, *n* = 6).The 70% liver ischemia model was used for the groups of mice with HIRI. We collected the feces sample from the diabetic and normal groups at ZT0 and ZT12 (Figure 1 the red dotted box). for 16S rRNA gene sequencing analysis and the FMT experiment.

Experiment 2: The diabetic and normal mice were administered with ABX (neomycin sulfate (200 mg/kg), metronidazole (200 mg/kg), ampicillin (200 mg/kg), and vancomycin (100 mg/kg) via oral gavage once a day for 4 days to eliminate the gut microbiota and induce the pseudo germ-free mice. Then, hepatic ischemia–reperfusion injury surgery was performed in pseudo germ-free diabetic mice and normal glucose mice. The liver pathological score and the serum levels of ALT and AST were analyzed to evaluate liver injury.

Experiment 3: To explore the role of the gut microbiota in diabetic mice with HIRI, we conducted the FMT experiment. We evaluated the damage to the liver using the liver pathological score and the serum levels of ALT and AST. Experiment 2 and Experiment 3 were conducted at ZT0.

Figure 1 is the schematic illustration of the experimental design.

### 2.8. Statistical Analysis

All results are expressed as mean ± standard error of the mean (SEM). Statistical differences among groups were analyzed using a one-way ANOVA (followed by post hoc Tukey’s multiple comparisons tests) using SPSS 27.0 software (IBM) and GraphPad Prism 8 software (San Diego, CA, USA). The sample size (*n*) is listed in each figure legend. Differences were considered statistically significant when *p* < 0.05.

## 3. Results

### 3.1. Diurnal Difference Does Not Affect HIRI in Diabetic Mice, and Hyperglycemia Aggravates HIRI

**Figure 2:**
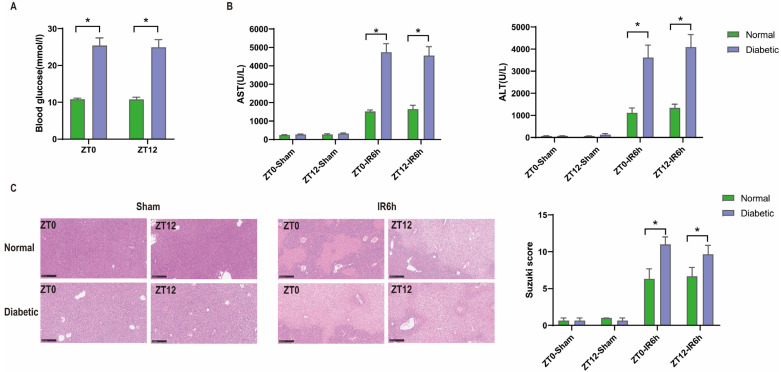
Hyperglycemia aggravates HIRI in mice, but without diurnal variation. (**A**) Glucose concentrations before the start of ischemia (*n* = 6). (**B**) Serum transaminase levels after 6 h of reperfusion (or sham) of diabetic group and normal group (*n* = 6). (**C**) HE staining and Suzuki scores (*n* = 3). Scale bar: 250 μm. One-way ANOVA with Bonferroni’s multiple comparisons test. * *p* < 0.05.

We established a diabetic model with significantly higher levels of blood glucose than the normal group. The data are presented in Figure 2A. Then, ischemia–reperfusion injury was performed in both the diabetic and normal groups. Liver damage, including serum transaminase levels (alanine aminotransferase (ALT) and aspartate aminotransferase (AST)), and histopathologic changes (Suzuki score) in HIRI were markedly exacerbated in the diabetic group when compared with those in the normal group after ischemia–reperfusion injury, while no difference was observed after the sham operation.

But there were no diurnal variations in the blood glucose levels, liver pathological score, or serum ALT/AST among these groups. These results demonstrated that hyperglycemia aggravates HIRI in mice, but without diurnal variation (Figure 2B,C).

### 3.2. Hepatic Ischemia–Reperfusion Injury Aggravated in Pseudo Germ-Free Mice

**Figure 3:**
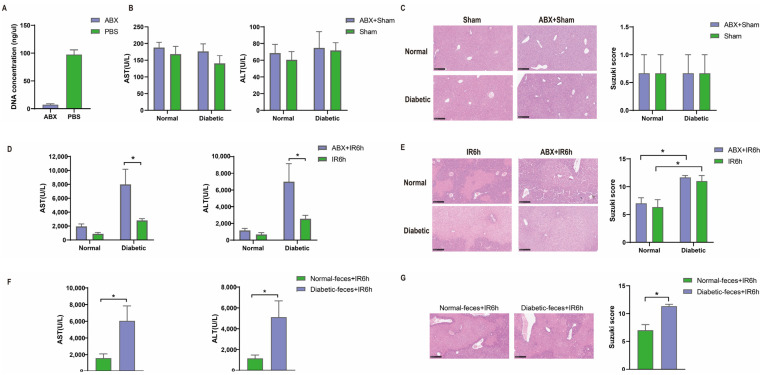
Diabetic fecal microbiota transplantation aggravated hepatic ischemia–reperfusion injury at ZT0. (**A**) Mice were administered ABX via oral gavage once daily for 4 days. The DNA concentration of the mice with ABX pretreatment (*n* = 6). (**B**,**C**) Serum transaminase levels of diabetic group and normal group with ABX pretreatment, HE staining, and Suzuki scores (*n* = 3). (**D**,**E**) Serum transaminase levels after 6 h of reperfusion of diabetic group and normal group with ABX pretreatment (*n* = 7), HE staining, and Suzuki scores (*n* = 3). (**F**,**G**) Serum transaminase levels after 6 h of reperfusion of FMT mice (*n* = 7), HE staining, and Suzuki scores (*n* = 3). * *p* < 0.05. Student’s *t* test or one-way ANOVA with Bonferroni’s multiple comparisons test.

The gut microbiome plays an important role in human metabolic processes. Thus, we conducted ABX (metronidazole–neomycin–vancomycin–ampicillin) pretreatment. We tested the DNA concentration of the feces from the mice with ABX pretreatement and it was significantly reduced, indicating that the gut microbiota was significantly reduced (Figure 3A). No difference was observed in terms of liver injury in the diabetic group and normal group with ABX pretreatment under sham operations. But oral ABX pretreatment enhanced liver injury after HIRI in the groups, and HIRI in the diabetic group was more apparent (*p <* 0.05, Figure 3D,E), indicating that the gut microbiota participates in the regulation of HIRI in diabetic mice. 

### 3.3. Diabetic Fecal Microbiota Transplantation Aggravates Hepatic Ischemia–Reperfusion Injury

When we conducted the FMT experiment, the diabetic mice with feces from the diabetic group showed aggravated HIRI compared with feces from the normal glucose group (Figure 3F,G). Thus, the gut microbiota from diabetic mice had harmful effects on HIRI compared with the gut microbiota from the normal glucose mice.

### 3.4. Alterations in the Gut Microbiota Composition between the Diabetic and Control Mice

**Figure 4:**
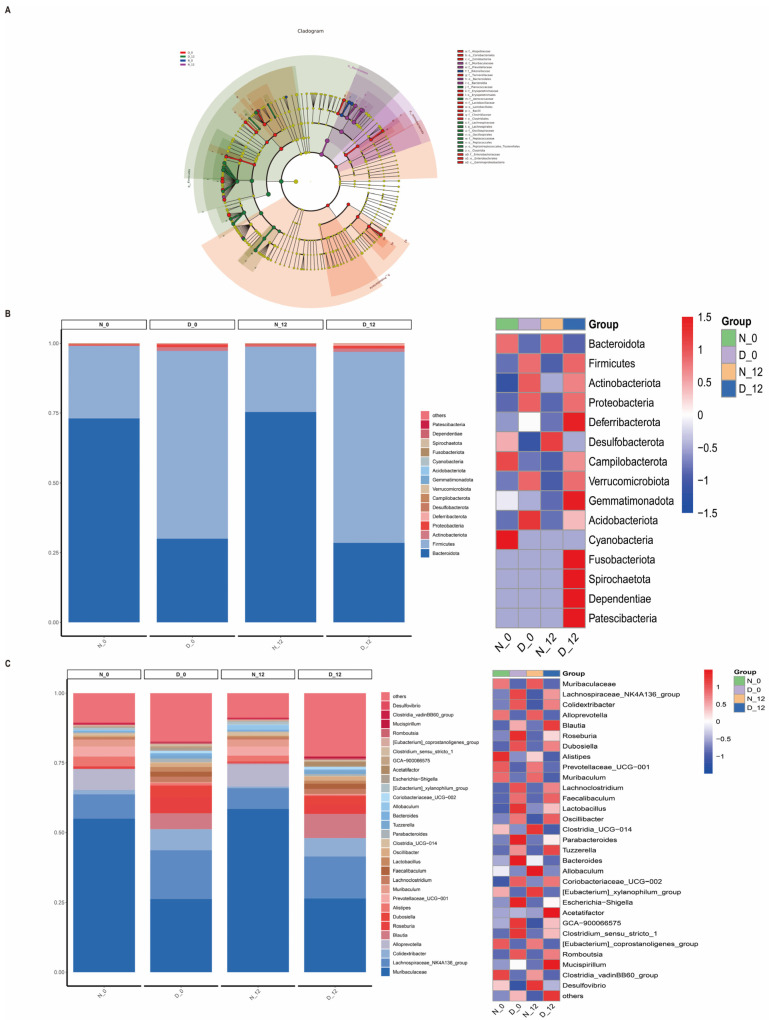
Overall microbial composition of four kinds of feces samples were different at the phylum and genus level. (**A**) Linear discriminant analysis effect size (LEfSe) analysis. (**B**) Comparisons of relative abundance of gut microbiota at the phylum and (**C**) genus level in the feces (*n* = 6). D-0 (diabetic-ZT0), D-12 (diabetic-ZT12), N-0 (normal-ZT0), N-12 (normal-ZT12).

**Figure 5:**
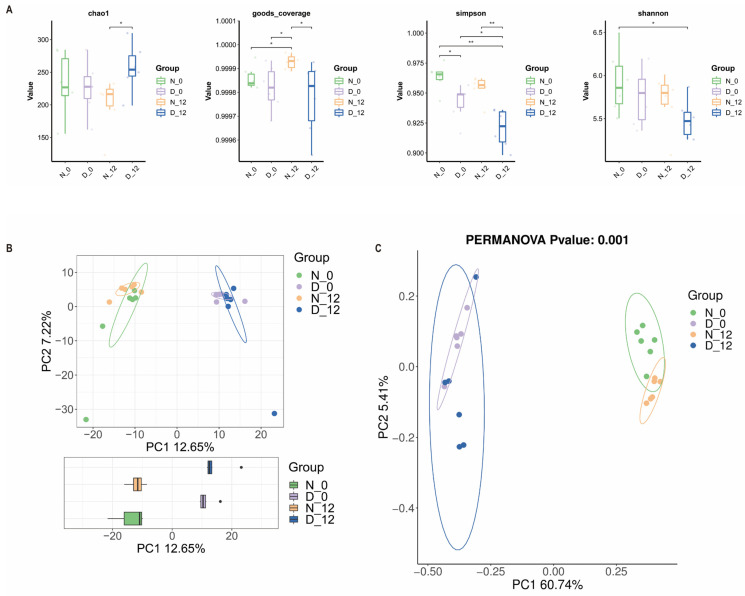
Alterations in the gut microbiota composition between the diabetic and control mice. Alpha diversity (**A**) and beta diversity, including PCA (**B**) and PCOA (**C**), in gut microbiota between the four groups, * *p* < 0.05, ** *p* < 0.01. D-0 (diabetic-ZT0), D-12 (diabetic-ZT12), N-0 (normal-ZT0), N-12 (normal-ZT12).

To explore the regulatory mechanism of the gut microbiota, we examined the compositional differences between the diabetic group and the normal group. In addition, we explored whether there were diurnal variations in the gut microbiota composition of diabetic mice. Thus, we performed 16S rRNA gene sequencing analysis for feces from the two groups at ZT0 and ZT12. Based on the linear discriminant analysis effect size (LEfSe) analysis, 29 discriminative features were identified (Figure 4A). Figure 4B shows that the overall microbial composition of four kinds of feces samples were different at the phylum and genus level. We compared the α-diversity of the microbiota between the diabetic feces and normal feces samples using the chao1, goods-coverage, Shannon, and Simpson indices, and they demonstrated a significant difference (Figure 5A). There were also significant differences among the beta diversity of four groups as measured using the PCA and PCoA (Figure 5B,C).

**Figure 6:**
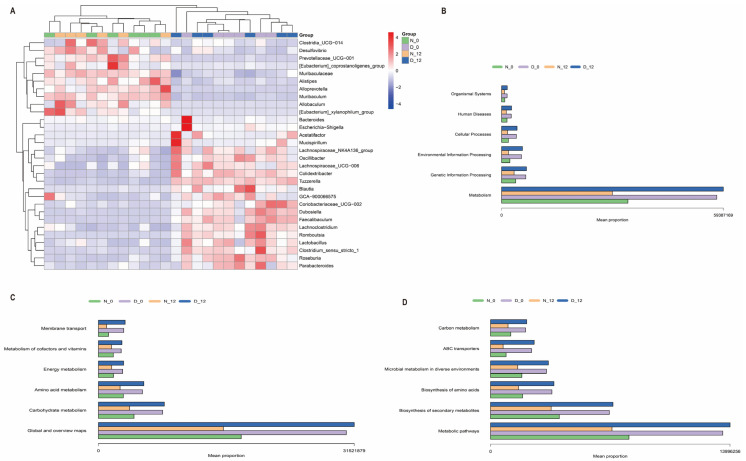
KEGG pathway database in the functional composition. (**A**) Heatmap of differential species at the genus level. (**B**–**D**) KEGG analysis, Level 1, 2, and 3.

Thirty differentially expressed species were found, with Muribaculaceae, Lachnospiraceae_NK4A136_group, Colidextribacter, Alloprevotella, Blautia, Roseburia, Dubosiella, Alistipes, Prevotellaceae_UCG−001, and Muribaculum being the top ten among the different groups (Figure 6A). Furthermore, the KEGG pathway database was utilized to elucidate the differences in functional composition. At Level 1, we observed that the higher abundance difference in the diabetic group was associated with metabolism, genetic information processing, etc. At Level 2, the proportion of sequences was related to global and overview maps, carbohydrate metabolism, amino acid metabolism, etc. At Level 3, they were significantly enriched in the metabolic pathways, biosynthesis of secondary metabolites, biosynthesis of amino acids, etc. (Figure 6B–D).

## 4. Discussion

The present study investigated changes in the gut microbiota and circadian rhythm oscillation of hepatic ischemia–reperfusion injury in diabetic mice, as assessed by fecal microbiota transplantation and 16S rRNA gene sequencing. The principal findings are as follows: (1) hyperglycemia aggravated HIRI in diabetic mice, but there was no diurnal variation seen in the diabetic HIRI. (2) There were significant alterations in the gut microbiota composition between the diabetic and control mice. (3) Gut microbiota transplantation from diabetic mice had obvious harmful effects on HIRI.

The circadian rhythm plays a crucial role in diabetes metabolism. A recent study has also found that the central circadian clock could regulate the diurnal rhythm of hepatic insulin sensitivity [20]. The liver is a major metabolic organ as well as the main peripheral circadian organ, which has an important role in regulating glucose homeostasis. Acetaminophen is thought to exhibit hepatotoxicity variation over a 24 h period, which is markedly increased at night when compared with the morning in mice [21]. The glucose metabolism in the liver also showed rhythmic oscillations [22]. Another recent study also reported diurnal variation in HIRI in mice [14], which is closely related to the diurnal composition of the gut microbiota. Therefore, our study firstly aimed to explore diurnal variation in HIRI in diabetic mice. Interestingly, diabetic hepatic ischemia–reperfusion injury was not affected by diurnal variation in our study. There was also no circadian rhythm difference in the mice with normal blood glucose. More experiments and samples are needed to verify whether there are circadian differences in the HIRI of mice. In addition, we observed that hyperglycemia aggravated HIRI in diabetic mice because of diabetes-related microcirculatory disease and the inflammatory response, which is consistent with earlier studies [23,24]. Diabetic mice have also been shown to be more susceptible to liver ischemia.

Moreover, hyperglycemia is known to affect the expression of core clock genes in livers [25]. Further reports have indicated that the diurnal oscillations of the clock gene BMAL1 were significantly attenuated in diabetic hearts [26]. The relationship between hyperglycemia and liver clock genes has a complex effect on the outcome of hepatic ischemia–reperfusion injury. Our results are only preliminary, and the effects on liver ischemia still need to be further investigated. 

When we explored the potential role of the gut microbiota in diabetic mice suffering from hepatic ischemia–reperfusion injury, the subsequent HIRI of the pseudo germ-free mice in the experiment confirmed the role of the gut microbiota in the liver injury of diabetic mice. In the two groups, pretreatment with ABX was associated with more severe liver injury after 6 h of reperfusion, but the HIRI in the diabetic group was more pronounced (*p* < 0.05). This could be due to the fragile nature of diabetic mice [27] and the role of the gut microbiota, which is amplified in hepatic ischemia–reperfusion injury, resulting in the differences found. Therefore, the group with normal blood glucose levels only showed a trend toward increased damage. As the liver injury of ABX + IR6h and IR6h in the diabetic groups was severe, the Suzuki scores were close to the maximum of 12, so there was no statistical difference between both groups shown in Figure 3E. If more detailed liver injury scores were available, differences might occur. AST and ALT levels have been the gold standard for the detection and quantification of liver injury. In Figure 3D, the ALT and AST levels of the ABX + IR6h group were obviously higher than the levels of the IR6h group. So, we believe that the gut microbiota participates in the regulation of HIRI in diabetic mice.

At present, fecal microbiota transplantation is a relatively mature experimental technology, and it is also applied to diagnose, ease, treat, or prevent disease or influence the structure or function of the body [28]. Current evidence deems FMT as a generally safe therapeutic method with few adverse effects. Many studies also provide relevant data and evidence of the success of the fecal microbiota transplantation experiment [29,30]. So, we conducted the FMT experiment to examine the role of intestinal microbiota in diabetic mice with HIRI. From the result of the FMT experiment, the gut microbiota of the diabetic group had harmful effects on HIRI compared with the intestinal microflora of the normal group.

We compared the feces from both the normal and diabetic groups at ZT0 and ZT12 using 16S rRNA gene sequencing analysis. The results showed that the overall microbial composition of normal and diabetic feces samples was distinctly different at the phylum and genus levels. Moreover, the alpha diversity of the gut microbiota exhibited a significant difference between the normal and diabetic mice at 8:00 a.m. at 20:00 p.m., while the beta diversity changed significantly between the diabetic and normal mice, although not significantly, over time. Alpha diversity is the species diversity present within a group. Beta diversity is represented by the species diversity between any two groups; it is on a larger scale, and looks to compare the species diversity between two separate entities that are often divided by environment. Their calculations and their nature are different [31]. Circadian rhythms may affect the number of species in a group, but the difference in species composition between the two groups is not obvious. But the species diversity of diabetic mice is significantly different from normal mice. At the genus level, the abundance of *Lachnospiraceae_NK4A136_group*, *Colidextribacter*, *Blautia*, *Roseburia*, *Dubosiella*, *Lachnoclostridium*, *Faecalibaculum*, *Lactobacillus*, and *Oscillibacter* was significantly higher in the diabetic group when compared to the normal group. A study demonstrated that the *lactobacillacea* was enriched in type 2 diabetes patients and was the best predictor among all microbial strains [32]. Several studies have reported that a high-fat diet is associated with an increase in the phylum Firmicutes and a relatively reduced abundance of the phylum Bacteroidetes [33,34]. Diabetic mice were fed a high-fat (proteins 20%, carbohydrates as sucrose, maltodextrin 20%, lipids 60% of total kcal) Research Diet for one month before being injected with streptozotocin. Our result is consistent with that of others [35], since the composition of the feces samples from ZT0 and ZT12 was subtly different. This could explain the reason for the absence of circadian rhythm differences in liver ischemia–reperfusion injury. Mechanistic studies on rodents identified that hyperglycemia may increase intestinal barrier permeability and alter tight junction integrity through the GLUT2-dependent transcriptional reprogramming of intestinal epithelial cells [36]. The gut microbiota regulate the production of different metabolites. Microbial metabolites may be the potential underlying mechanism. In further studies, we will perform metabolomics analysis of feces to explore the potential mechanism. We would like to conduct the transplantation of certain bacteria to explore the possible potential mechanisms of action. The gut microbiome has demonstrated a massive and complex inter-individual variation, and the changes in the composition of the gut microbiota also interact with the occurrence and development of diabetes [37]. Our results also indicated significant differences in the composition of the microbiome between the diabetic and normal glycemic groups. Therefore, this study provides us with an indication that we should pay attention to the possible adverse events of preoperative gut microbiota disturbance and consider some appropriate corrective measures.

The differences in the gut microbiota and its metabolites have also been shown to play an important role in the ischemia–reperfusion (I/R) injury of other organs. The gut microbiota metabolite capsiate was found to enhance Gpx4 expression and inhibit ferroptosis by activating TRPV1 in intestinal I/R injury, providing a potential avenue for the management of intestinal I/R injury [38]. A study indicated that the gut microbiota restricts NETosis in acute mesenteric I/R injury, and it could enhance neutrophil recruitment to ensure immunovigilance related to the TLR4/TRIF pathway [39]. The gut microbiome is associated with the prognosis of intestinal I/R. Lactobacillus murinus could promote the release of IL-10 from macrophages through TLR2 to reduce intestinal I/R injury, and it may be a potential mechanism for *L. murinus* to alleviate intestinal I/R injury in mice [40]. Different from the above two studies, the gut microbiota aggravated cardiac I/R injury. The gut microbiota regulates the formation of NETs, which directly leads to the apoptosis of cardiomyocytes and myocardial microvascular endothelial cells. The gut microbiota, migrated to the blood, stimulates the formation of NETs after cardiac I/R [41]. Studies have also shown that the gut microbiota could profoundly influence the brain function of the host. The composition of the gut microbiota in mice with cerebral I/R injury could influence the pathobiology of cerebrovascular diseases, including brain functional connectivity, neuroinflammation, and animal behavior [42]. Studies reported that the significant changes in the composition of gut microbiota caused byrenal IRI, which is closely related to the prognosis of renal IRI [43].

There are some limitations to our study. We did not conduct the FMT experiment with feces samples at ZT0 and ZT12. because we did not identify a diurnal variation in HIRI in diabetic mice. In addition, we did not delve into the specific gut microbial metabolites, as they may exert effects on hepatic I/R injury. The effect of gut microbial metabolites on hepatic I/R injury requires further investigation.

In summary, our findings revealed that hyperglycemia aggravates HIRI in diabetic mice, specifically in that intestinal microbes participated in the regulation of HIRI. We reported the differences in the microflora composition in diabetic and normal mice at different time points, hoping to provide some useful information for future research.

## Data Availability

All relevant data supporting reported results are available from the corresponding author on reasonable request.
